# Genomic identification of cotton *SAC* genes branded ovule and stress-related key genes in *Gossypium hirsutum*

**DOI:** 10.3389/fpls.2023.1123745

**Published:** 2023-02-03

**Authors:** Ma Shuya, Liu Le, Shi Huiyun, Gu Yu, Li Yujun, Ghulam Qanmber

**Affiliations:** ^1^ Zhengzhou Research Base, State Key Laboratory of Cotton Biology, School of Agricultural Sciences, Zhengzhou University, Zhengzhou, Henan, China; ^2^ State Key Laboratory of Cotton Biology, Institute of Cotton Research, Chinese Academy of Agricultural Sciences, Anyang, Henan, China; ^3^ College of Agronomy, Shenyang Agricultural University, Shenyang, China; ^4^ Engineering Research Centre of Cotton, Ministry of Education, Xinjiang Agricultural University, Urumqi, China

**Keywords:** cotton, phylogenetic analysis, SAC genes, multiple synteny, flower, ovule, abiotic stresses

## Abstract

*SAC* genes have been identified to play a variety of biological functions and responses to various stresses. Previously, *SAC* genes have been recognized in animals and *Arabidopsis*. For the very first time, we identified 157 *SAC* genes in eight cotton species including three diploids and five tetraploids with 23 *SAC* members in *G. hirsutum*. Evolutionary analysis classified all cotton *SAC* gene family members into five distinct groups. Cotton *SAC* genes showed conserved sequence logos and WGD or segmental duplication. Multiple synteny and collinearity analyses revealed gene family expansion and purifying selection pressure during evolution. *G. hirsutum SAC* genes showed uneven chromosomal distribution, multiple exons/introns, conserved protein motifs, and various growth and stress-related *cis*-elements. Expression pattern analysis revealed three *GhSAC* genes (*GhSAC3*, *GhSAC14*, and *GhSAC20*) preferentially expressed in flower, five genes (*GhSAC1*, *GhSAC6*, *GhSAC9*, *GhSAC13*, and *GhSAC18*) preferentially expressed in ovule and one gene (*GhSAC5*) preferentially expressed in fiber. Similarly, abiotic stress treatment verified that *GhSAC5* was downregulated under all stresses, *GhSAC6* and *GhSAC9* were upregulated under NaCl treatment, and *GhSAC9* and *GhSAC18* were upregulated under PEG and heat treatment respectively. Overall, this study identified key genes related to flower, ovule, and fiber development and important genetic material for breeding cotton under abiotic stress conditions.

## Introduction

Phospholipids group that is different from other phospholipids based on the existence of a phosphate group of phosphatidylinositol (PI) are known as Phosphoinositides. Phosphoinositides exist in seven forms and are important for the release of intracellular calcium and the activation of protein kinase C (Toker, 1998). In animals and yeast (*Saccharomyces cerevisiae*), phosphoinositides play a key role in biological mechanisms including maintenance of vacuole morphology, actin cytoskeleton organization, vesicle trafficking, regulation of lipid storage, and proteins activation (Takenawa and Itoh, 2001). SAC proteins have been categorized into two groups based on protein sequences except for SAC domains (Hughes et al., 2000). In one group SAC domain is found in N-terminal and is linked to type II 5-phosphatase located in C-terminal. The SAC domain of the second group is associated with the C-terminal with unknown domains. In the second group, protein’s C-terminal regions are different in length from amino acid sequences. SAC domains have seven conserved motifs and about a length of 400 amino acids. SAC domains are important for phosphatase activities (Guo et al., 1999; Hughes et al., 2000).

In plants, *SAC* genes have been identified to play a variety of functions including pollen tube growth, vesicle trafficking, osmotic regulation, and responses to hormonal treatments and various stresses (Zhong and Ye, 2003; Wang et al., 2017; Xiong et al., 2019). Genome analysis indicated a large number of *SAC* genes in *Arabidopsis*. However, limited studies investigated the functions of *SAC* genes in plants. In *Arabidopsis*, FRA7 encodes a SAC protein and plays important functions as cell morphogenesis was altered in the *fra7* mutant (Erdman et al., 1998). Further, defective cell morphogenesis and cell wall biosynthesis was observed by truncated *SAC1* in *Arabidopsis* (Zhong et al., 2005). *Arabidopsis SAC2*, *SAC3*, *SAC4*, and *SAC5* have recognized tonoplast-associated enzymes and play functions in vacuolar morphology. *SAC6* showed high expression in flowers with induced expression by salinity stress (Zhong and Ye, 2003), and *SAC7* showed involvement in root hair growth in *Arabidopsis* (Thole et al., 2008).

Cotton is a chief fiber crop and a model to study polyploidy, species evolution, cellulose biosynthesis as well as cell wall development (Senchina et al., 2003). Fiber development is an intricate process that entails several plant hormones such as auxin, gibberellins (GAs), ethylene, and brassinosteroids (Seagull and Giavalis, 2004; Samuel Yang et al., 2006; Ali et al., 2021; Wu et al., 2021a). The genus *Gossypium* contains about 45 diploid species and seven tetraploid cotton species (Li et al., 2019; Yang et al., 2020). All diploid and tetraploid *Gossypium* species constitute a single monophyletic group originating from a common ancestor around 5–10 million years ago (mya). Among seven allopolyploid cotton species, including *G. hirsutum* (AD1), *G. barbadense* (AD2), *G. tomentosum* (AD3), *G. mustelinum* (AD4), *G. darwinii* (AD5), *G. ekmanianum* (AD6), and *G stephensii* (AD7), *G. mustelinum* may serve as the basal clade, with AD1 and *G. tomentosum* forming the second clade, whereas AD2 and *G. darwinii* form the third clade (Huang et al., 2021). Hybridization among A genome having similar genomic characteristics of *G. herbaceum* (A1) or *G. arboreum* (A2) and a D genome having similar genomic characteristics of *G. raimondii* (D5) with subsequent polyploidization gave rise to seven tetraploid cotton species including *G. hirsutum* and *G. barbadense* around 1-2 mya (Wendel and Cronn, 2003; Malik et al., 2018). With the improvements in sequencing and assembly of cotton genomes (Paterson et al., 2012; Du et al., 2018) it is possible to perform a complete study of cotton gene families. Functions of many *SAC* genes have been identified in *Arabidopsis* but there is no previously published study of *SAC* genes in cotton.

We comprehensively identified and characterized the *SAC* gene family members in three diploid species (*G. herbaceum*, *G. arboreum*, and *G. raimondii*), and five tetraploid species (*G. hirsutum*, *G. darwinii*, *G. tomentosum*, *G. barbadense*, and *G. mustelinum*) of cotton. The evolutionary relationship among cotton *SAC* genes was determined by phylogenetic analysis, gene structure, conserved motifs, and sequence logos analysis. Next, multiple synteny analysis and collinearity analysis with nonsynonymous (*Ka*) and synonymous (*Ks*) substitution ratios (*Ka/Ks* ratios) were estimated. Moreover, functions of *GhSAC* genes were observed by promoter *cis*-elements analysis, tissue specific expression patterns analysis, and the expression of *GhSAC* genes after abiotic stress treatments.

## Materials and methods

### Identification of cotton *SAC* genes

The gene sequences, protein, cDNA and gene annotation, and genome files (gff) of *G. herbaceum* (WHU, version 1.0), *G. arboreum* (ICR, version 1.0), *G. raimondii* (JGI, version 1.0), *G. hirsutum* (ICR, ZM24 version 1.0), *G. barbadense* (HAU, version 1.0), *G. tomentosum* (HGS, version 1.0), *G. mustelinum* (HGS, version 1.0) *G. darwinii* (HGS, version 1.0) were obtained from the CottonFGD database (Zhu et al., 2017). The identified SAC protein sequences in *Arabidopsis* (Zhong and Ye, 2003) were used to find the *SAC* genes in observed cotton species by Local BLASTP search. The identified *SAC* genes were also confirmed by HMM (hidden Markov model) profile obtained from the Pfam (PF02383) database (Finn et al., 2016), PROSITE (PS50275), and Interproscan 63.0 (IPR002013) (http://www.ebi.ac.uk/InterProScan/) (Jones et al., 2014). Identified SAC domains containing proteins were further confirmed by NCBI Batch-CD search (https://www.ncbi.nlm.nih.gov/Structure/bwrpsb/bwrpsb.cgi). We also compared the results of *GhSAC* genes identified from *G. hirsutum* (ICR, ZM24 version 1.0) with HAU, JGI, NAU, and ICR (TM-1 version 1.0) and found no difference.

### Phylogenetic and sequence logos analysis of *SAC* genes

For the phylogenetic analysis, amino acid sequences from *G. arboreum*, *G. hirsutum*, *G. herbaceum*, *G. raimondii*, *G. darwinii*, *G. barbadense*, *G. mustelinum*, and *G. tomentosum* were aligned by Clustal (Larkin et al., 2007). MEGA 7.0 with ML (Maximum likelihood) method and 1000 bootstrap value was used to generate a tree. For sequence logos analysis, we aligned the SAC protein sequence of *G. arboreum*, *G. hirsutum*, *G. herbaceum*, *G. raimondii*, *G. darwinii*, *G. barbadense*, *G. mustelinum*, and *G. tomentosum* by Clustal X 2.0 (Thompson et al., 1997). Sequence logos were constructed using the online tool WEBLOG (Crooks et al., 2004).

### Gene structure, motif distribution, and promoter *cis*-elements analysis

For gene structure analysis, ClustalW was used to align GhSAC protein sequences, and MEGA 7.0 (Kumar et al., 2016) was used to construct an NJ tree. The exon/intron pattern was predicted by GSDS 2.0 (Hu et al., 2010). Protein motif distribution patterns were determined by using the MEME program (http://meme-suite.org/tools/meme) (Bailey et al., 2006) as stated before (Qanmber et al., 2018). Next, for the analysis of *cis*-elements, 2000 bp promoter sequences of *GhSAC* were obtained from CottonFGD (Zhu et al., 2017). The *GhSAC* promoter *cis*-elements were predicted using the PlantCARE (Plant Cis-Acting Regulatory Element) database (Lescot et al., 2002).

### Chromosomal location, gene duplication, and multiple synteny analysis

To investigate the chromosomal location of *GhSACs*, gff-files of cotton genome annotation data were extracted from the CottonGen database (ftp://ftp.bioinfo.wsu.edu/species/Gossypium_hirsutum/NAU-NBI_G) and genes were mapped by MapInspect software (Jia et al., 2018) on their chromosomes. For gene duplication analysis CIRCOS (Krzywinski et al., 2009) and figure was made by TBtools (Chen et al., 2020). Next, we used PAL2NAL (Suyama et al., 2006) and PAML package (Yang, 2007) to calculate *Ka/Ks* values.

### RNA extraction and qRT-PCR analysis

Leaf samples of various tissues were collected and RNAprep Pure Plant Kit (TianGen, Beijing, China) was used to obtain RNA. RNA was converted into cDNA by EasyScript Allin- First-strand cDNA synthesis SuperMix for RTqPCR kit (TransGen, Beijing, China) and used as a template for qRT-PCR. TransStart Top Green qPCR SuperMix (TransGen, Beijing, China) was used to perform qRT-qPCR in LightCycler 480 (Roche, Basel, Switzerland). Each experiment was conducted in three biological replicates and GhHis3 (AF024716) was used for the normalization of gene expression. Primers used in this study are given in **Supplementary Table S1** and qRT-PCR analysis was performed by the 2 −ΔCT method (Livak and Schmittgen, 2001).

## Results

### Genomic identification of *SAC* genes

In this study we identified 157 *SAC* genes in eight *Gossypium* species including 10 genes in *G. herbaceum*, 11 genes in *G. arboreum*, 12 genes in *G. raimondii*, 23 genes in *G. hirsutum*, 26 genes in *G. mustelinum*, 25 genes in *G. barbadense*, 25 genes in *G. tomentosum* and 25 genes each in *G. darwinii* (**Supplementary Table S2**). Interestingly, D genome cotton *G. raimondii* contains more numbers of genes than A genome cotton *G. arboreum* or *G. herbaceum*. Similarly, tetraploid cotton species (*G. barbadense*, *G. hirsutum*, *G. mustelinum*, *G. tomentosum*, and *G. darwinii*) contained almost double the numbers of *SAC* genes than diploid cotton species (*G. arboreum*, *G. herbaceum*, and *G. raimondii*). Among tetraploid species, *G. hirsutum* showed fewer numbers of *SAC* genes, however, indicated the effects of hybridization and polyploidization in allotetraploid cotton species. All identified *SAC* gene family members were renamed according to the position on their corresponding chromosomes (**Supplementary Table S2**).

Next, we predicted the basic features of *SAC* genes in observed *Gossypium* species and presented them in **Table 1**. Results indicated that 10 *SAC* genes of *G. herbaceum* showed protein length ranges from 558-1631 amino acids (aa) with a mean length of 861.1aa, a median length of 831.5aa and a total length of 8611aa, and 0.0506% occupied a position in the genome. *G. arboreum SAC* genes showed protein length ranges from 597-1631aa with a mean length of 843.18aa, a median length of 828aa and total length of 9275aa, and 0.0575% occupied position in the genome. *G. raimondii SAC* genes showed protein length ranges from 188-1631aa with a mean length of 851.83aa, median length of 828.5aa, a total length of 10222aa, and 0.0681% occupied position in the genome. Allotetraploid cotton *G. hirsutum SAC* genes showed protein length ranges from 565-1631aa with a mean length of 868.17aa, the median length of 828aa, and total length of 19968aa and 0.0673% occupied a position in the genome.

**Table 1:** Features of cotton *SAC* genes.

Species	No. of SAC genes	Minimum Length (aa)	Maximum Length (aa)	Mean Length	Median Length	Total Length of all SAC genes (aa)	Occupied position in genome (%)
*G. herbaceum* (A1)	10	558	1631	861.1	831.5	8611	0.0506
*G. arboreum* (A2)	11	597	1631	843.18	828	9275	0.0575
*G. raimondii* (D5)	12	188	1631	851.83	828.5	10222	0.0681
*G. hirsutum* (AD1)	23	565	1631	868.17	828	19968	0.0673
*G. barbadense* (AD2)	25	187	1631	866.66	828	21661	0.0738
*G. tomentosum* (AD3)	25	205	1631	811.4	813	20285	0.0679
*G. mustelinum* (AD4)	26	163	1631	822.46	813	21384	0.0734
*G. darwinii* (AD5)	25	215	1631	868.56	827	21714	0.0727

Number, minimum, maximum, and median length, total length, and % occupied position in the genome for G. herbaceum, G. arboreum, G. raimondii, G. hirsutum, G. barbadense, G. tomentosum, G. mustelinum, and G. darwinii SAC genes was estimated.

### Phylogenetic analysis and sequence logos analysis of *SAC* gene family

**Figure 1:**
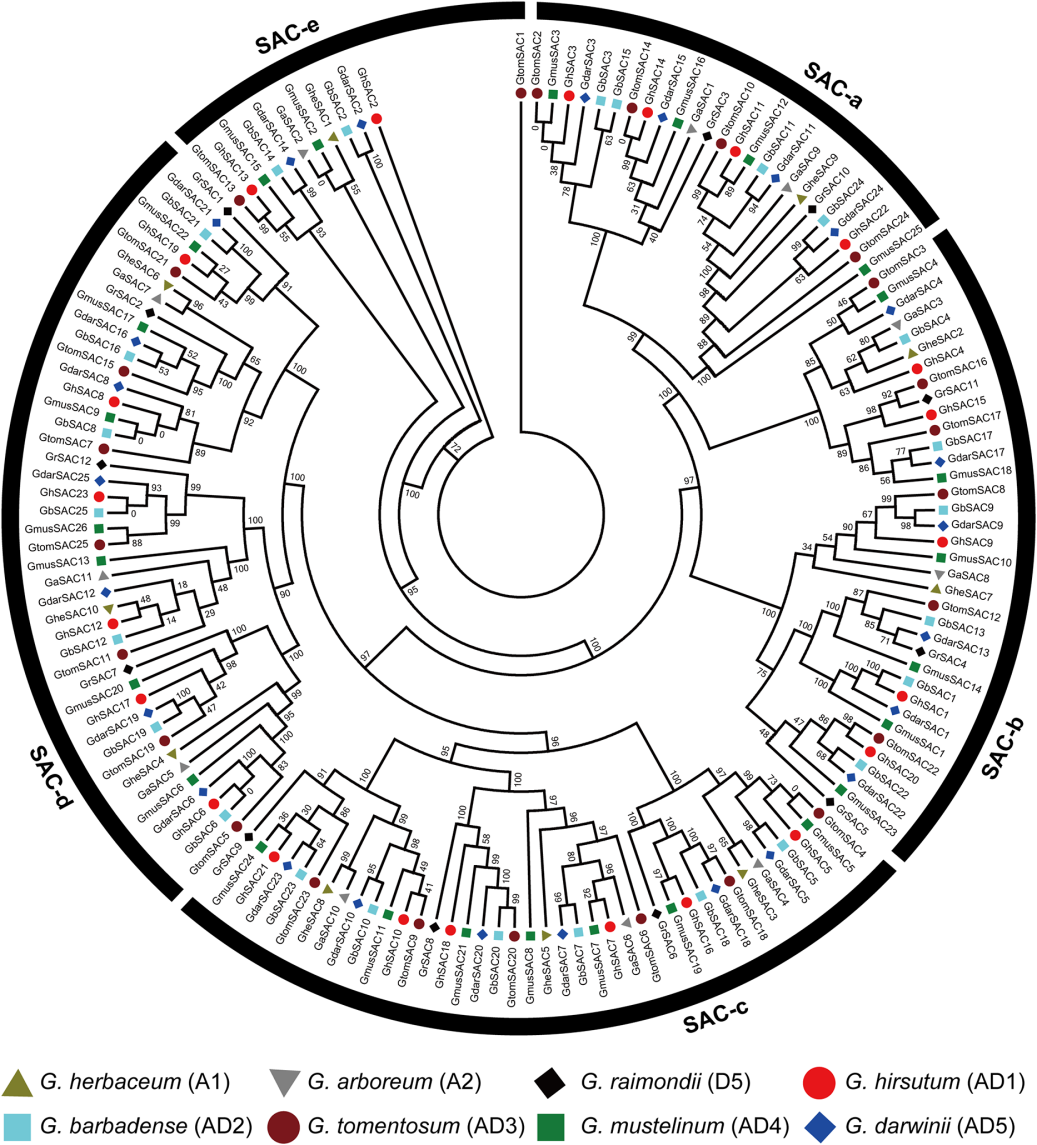
Phylogenetic analysis of cotton *SAC* genes. Phylogenetic tree among 157 *SAC* genes from three diploids (*G. herbaceum*, *G. arboreum*, and *G. raimondii*) and five tetraploids (*G. hirsutum*, *G. barbadense*, *G. tomentosum*, *G. mustelinum*, and *G. darwinii*) cotton species. The prefixes Ghe. Ga, Gr, Gh, Gb, Gt, Gm, and Gd represents *G. herbaceum*, *G. arboreum*, *G. raimondii*, *G. hirsutum*, *G. barbadense*, *G. tomentosum*, *G. mustelinum* and *G. darwinii* respectively.

To explore the evolutionary relationship of cotton *SAC* genes, all protein sequences were subjected to MEGA 7.0 software and a phylogenetic tree was constructed. To indicate the *SAC* genes from *G. arboreum*, *G. herbaceum*, *G. raimondii*, *G. hirsutum*, *G. barbadense*, *G. tomentosum*, *G. mustelinum*, and *G. darwinii*, the prefixes Ga, Ghe, Gr, Gh, Gb, Gt, Gm and Gd were used, respectively. The phylogenetic tree classified cotton *SAC* genes into five distinct groups SAC a-d (**Figure 1**). SAC-d and SAC-c were the largest groups containing 44 members each while SAC-e was the smallest with 12 members. SAC-b was the second largest group with 36 members. The phylogenetic tree displayed that most homologous *SAC* genes between diploids and tetraploids were closely clustered in the same group, indicating the expansion and evolutionary relationship of the *SAC* gene family. The phylogenetic tree indicated that groups SAC-a, SAC-b, SAC-c, and SAC-d contain *SAC* genes from eight observed cotton species while SAC-e lacks *G. raimondii* genes.

Further, the evolutionary pattern of *SAC* genes was observed in eight observed cotton species. Multiple sequence alignment of *G. arboreum*, *G. hirsutum*, *G. herbaceum*, *G. raimondii*, *G. darwinii*, *G. barbadense*, *G. mustelinum*, and *G. tomentosum SAC* genes was performed in MEGA 7.0 software and sequence logos were constructed (**Supplementary Figure S1**). Sequence logos of conserved amino acid residues among all the observed species were highly conserved (**Supplementary Figure S1A–H**). Sequence logos of conserved amino acid residues provide a better explanation of sequence identity.

### Gene duplication, multiple synteny, and collinearity analysis of *SAC* genes

To study the evolution and effects of hybridization and polyploidization, we identified the types of duplication of *SAC* genes in observed cotton species. Results identified that *G. arboreum*, *G. hirsutum*, *G. herbaceum*, *G. raimondii*, *G. darwinii*, *G. barbadense*, *G. mustelinum*, and *G. tomentosum SAC* genes showed WGD (whole genome duplication) or segmental duplication. However, one *SAC* gene from *G. raimondii*, two *SAC* genes from *G. barbadense*, two *SAC* genes from *G. tomentosum*, one *SAC* gene from *G. mustelinum*, and two *SAC* gene from *G. darwinii* showed the dispersed type of gene duplication. Additionally, one *SAC* gene from *G. tomentosum* and one *SAC* gene from *G. mustelinum* showed a singleton type of gene duplication (**Supplementary Table S3**).

**Figure 2:**
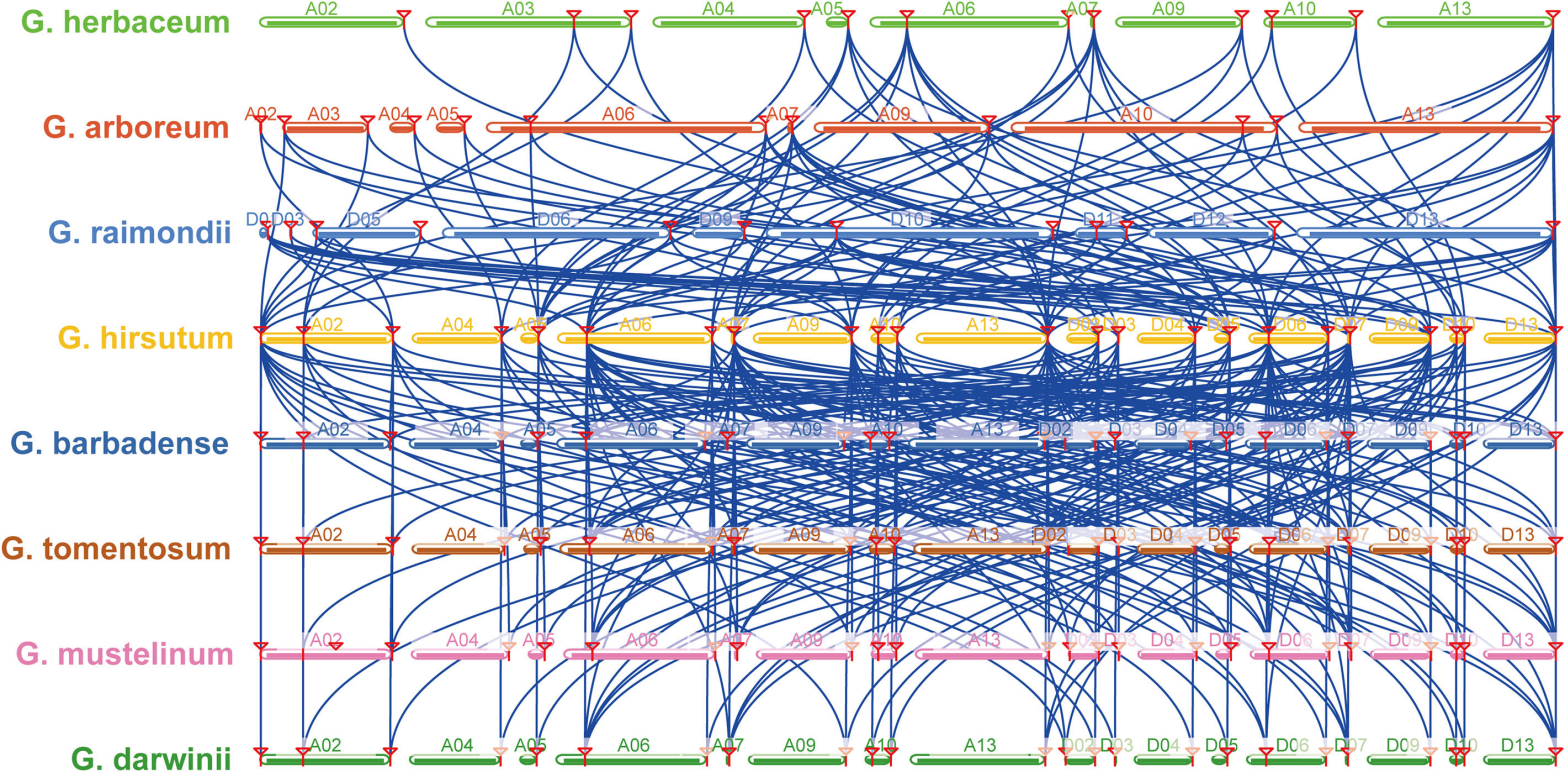
Multiple synteny analysis among cotton *SAC* genes. Multiple synteny analysis was used to show the orthologous relationship among *G. herbaceum*, *G. arboreum*, *G. raimondii*, *G. hirsutum*, *G. barbadense*, *G. tomentosum*, *G. mustelinum*, and *G. darwinii SAC* genes. Chromosomes of different cotton species were represented with different colors.

Multiple synteny analysis among *G. herbaceum*, *G. arboreum*, *G. raimondii*, *G. hirsutum*, *G. barbadense*, *G. tomentosum*, *G. mustelinum*, and *G. darwinii SAC* genes showed 41 orthologous gene pairs between *G. hirsutum* and *G. arboreum*, 43 between *G. hirsutum* and *G. herbaceum*, 44 between *G. hirsutum*, and *G. raimondii*, 65 between *G. hirsutum* and *G. barbadense*, 65 between *G. hirsutum* and *G. darwinii*, 64 between *G. hirsutum* and *G. mustelinum*, and 66 orthologous gene pairs between *G. hirsutum* and *G. tomentosum* (**Figure 2**; **Supplementary Table S4**). Further, the nonsynonymous and synonymous substitution ratios (*Ka/Ks* ratios) were estimated to find the type of selection pressure in these orthologous gene pairs during evolution. All homologous gene pairs between *G. hirsutum* and *G. herbaceum*, *G. hirsutum* and *G. arboreum*, *G. hirsutum* and *G. darwinii* showed *Ka/Ks* ratios less than 1. While all orthologous gene pairs showed *Ka/Ks* ratios less than 1 except one gene pair *G. hirsutum* and *G. raimondii*, one gene pair *G. hirsutum* and *G. barbadense*, one gene pair *G. hirsutum* and *G. mustelinum*, and two gene pair *G. hirsutum* and *G. tomentosum* (**Supplementary Table S4**).

**Figure 3:**
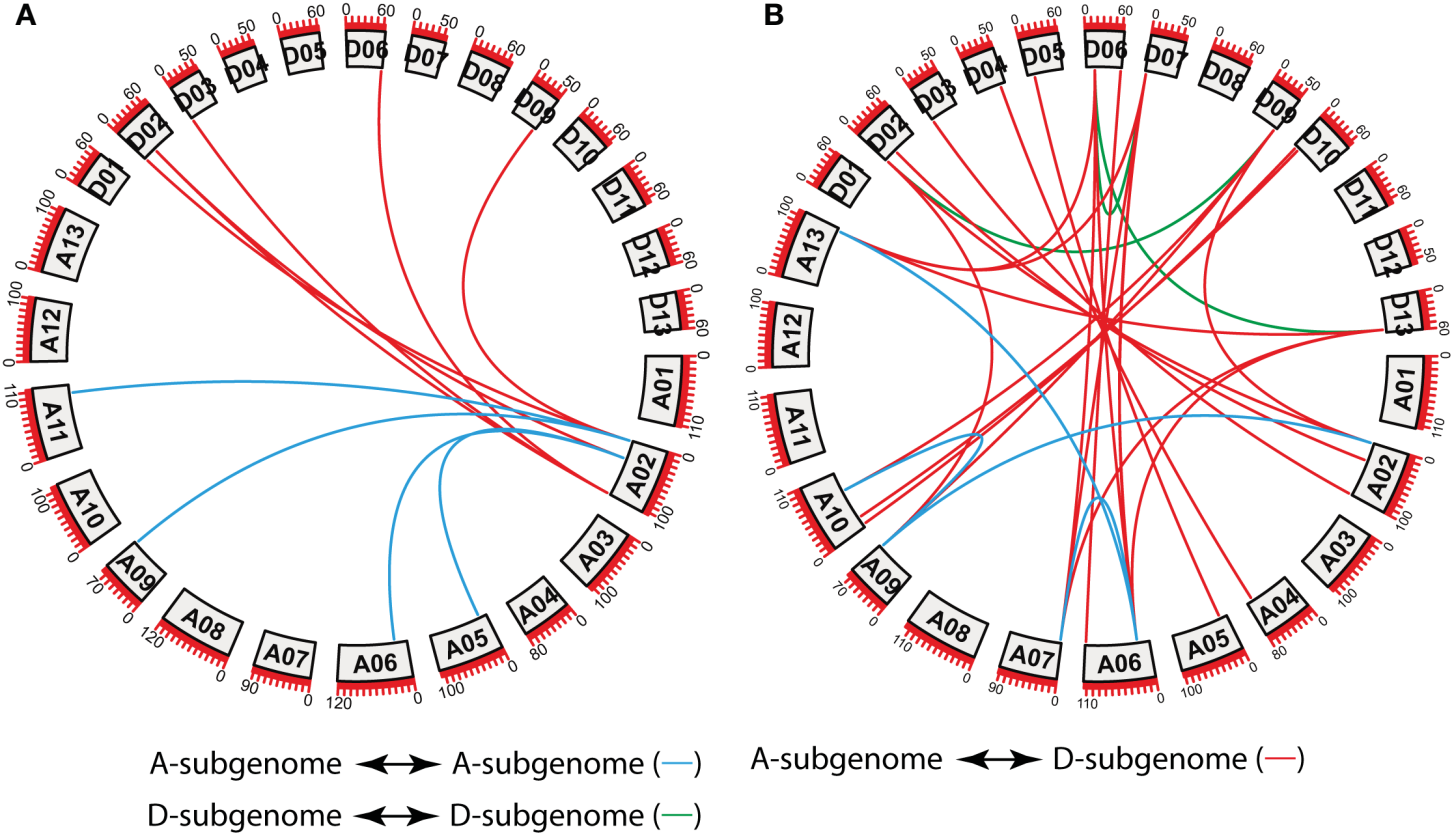
Collinearity analysis of *G*. *hirsutum* and *G*. *barbadense SAC* genes. **(A)** Collinearity analysis of *G*. *hirsutum SAC* genes. **(B)** Collinearity analysis of *G. barbadense SAC* genes. A01 to A13 represents A-subgenome chromosomes while D01 to D13 represents D-subgenome chromosomes. Homologous gene pairs between A- to A-subgenome were represented with blue lines, homologous gene pairs between A- to D-subgenome were represented with red lines, and homologous gene pairs between the D- to D-subgenome were represented with green lines.

To explore the locus relationships among the A- and D-subgenomes of *G. hirsutum* and *G. barbadense*, we performed a collinearity analysis (**Figure 3**). A total of 10 orthologous/paralogous pairs were found in *G. hirsutum* with *Ka/Ks* < 1 (**Figure 3A**; **Supplementary Table S5**). Similarly, a total of 28 orthologous/paralogous gene pairs were found in *G. barbadense* with *Ka/Ks* < 1 (**Figure 3B**; **Supplementary Table S5**). More precisely, all *GhSAC* genes showed *Ka/Ks* values <0.5 while the *Ka/Ks* values of 22 *GbSAC* genes were less than 0.5 while five genes showed *Ka/Ks* values greater than 0.5.

### Gene structure and protein motif analysis

To study the structural features, exon/intron and the protein motifs of *GhSAC* family genes were analyzed (**Supplementary Figure S2**). A NJ phylogenetic tree among *GhSAC* genes clustered according to the motif distribution pattern and exon-intron structure (**Supplementary Figure S2A**). The motif distribution pattern indicated the distribution of 10 motifs across the GhSAC proteins. GhSAC proteins with similar motif distribution patterns were closely clustered (**Supplementary Figure S2B**). Members of the same group have a similar motif distribution pattern, signifying that the motif distribution pattern is highly conserved and they might have identical functions. Next, the gene structure analysis indicated the distribution pattern of CDs, intron, and UTRs. Analysis indicated the presence of multiple introns in all observed *GhSAC* genes. However, the *GhSAC* genes with similar CDs, intron, and UTRs structures were found to make a representative clade in the phylogenetic tree (**Supplementary Figure S2C**).

### Chromosomal location and promotor *cis*-element analysis

Next, we inspected the location of *GhSACs* on chromosomes (**Supplementary Figure S3**). Findings showed that 23 *GhSAC* genes were distributed unevenly on 17 chromosomes. Out of 23 genes, 12 *GhSAC* genes were placed on the chromosomes of the A-subgenome while 11 *GhSAC* genes were located on the chromosomes of the D-subgenome. The maximum number of genes (three genes) were allocated on chromosome A02 of the A-subgenome and from D-subgenome the maximum genes were placed on the D10 chromosome (two genes). However, no gene was mapped on chromosome A01, A03, A08, A11 and A12 and D01, D08, D11, and D12 chromosomes.

**Figure 4:**
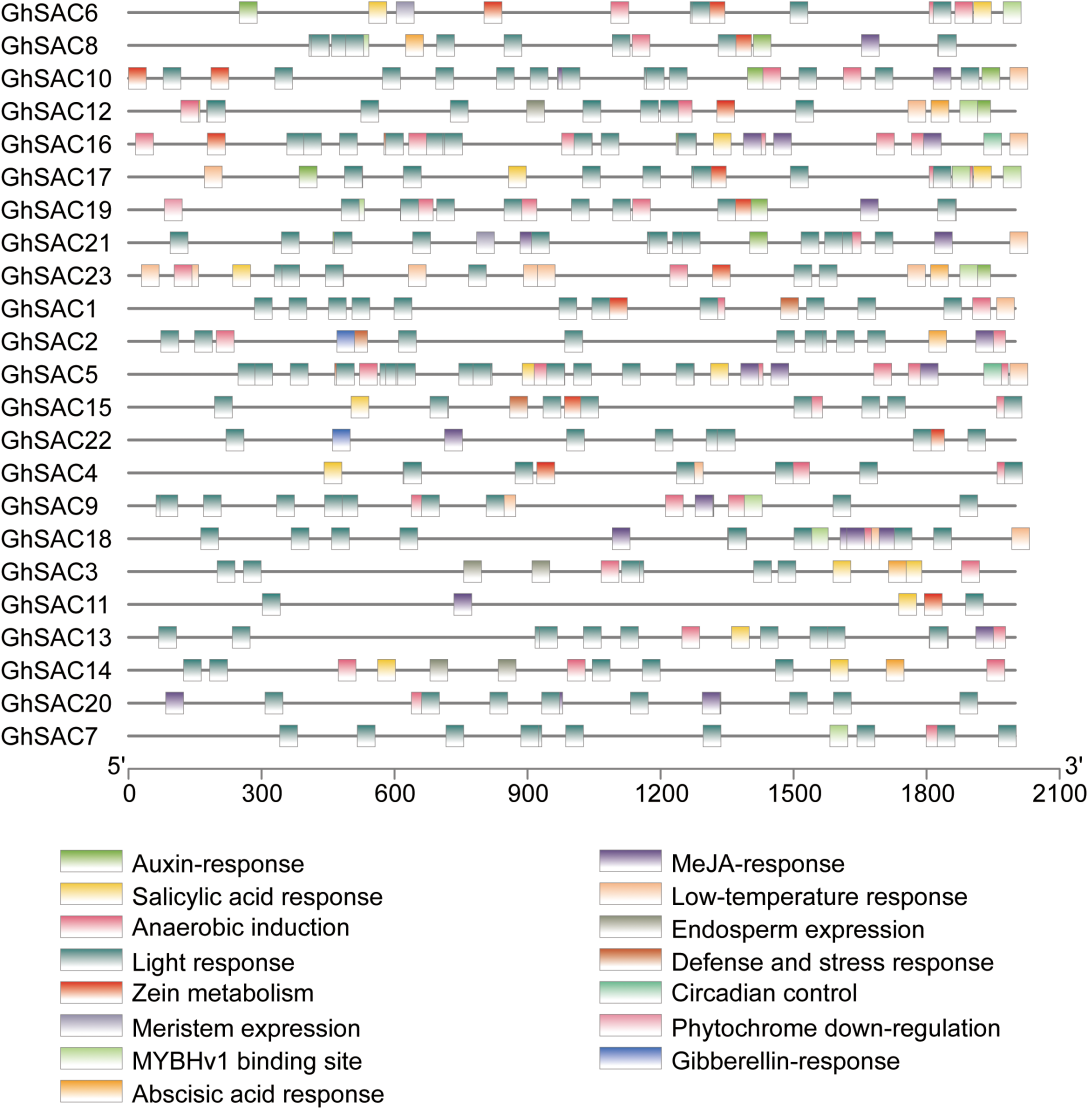
Promoter *cis*-element analysis of *GhSAC* genes. *G. hirsutum SAC* genes promoter region (2kb upstream from start codon) was used to explore *cis*-elements related to plant growth, abiotic stresses, and phytohormonal responses.

Furthermore, we used the PlantCARE database to identify the presence of *cis*-elements controlling the expression of the *GhSAC* genes. The results revealed that *GhSAC* gene promoters contain core *cis*-elements (**Figure 4**). The *GhSAC* genes promoter regions shared light-responsive boxes, zein metabolism, circadian control, anaerobic induction, and phytochrome downregulation elements. Further, stress-response elements including low-temperature response elements were present in the *GhSAC* promotor region. Growth-related elements including meristem expression and endosperm expression, hormone-related elements such as auxin response, salicylic acid response, abscisic acid response, MeJA response, and gibberellin response were found in the *GhSAC* promotor region.

### Tissue-specific expression pattern of *GhSAC* genes

**Figure 5:**
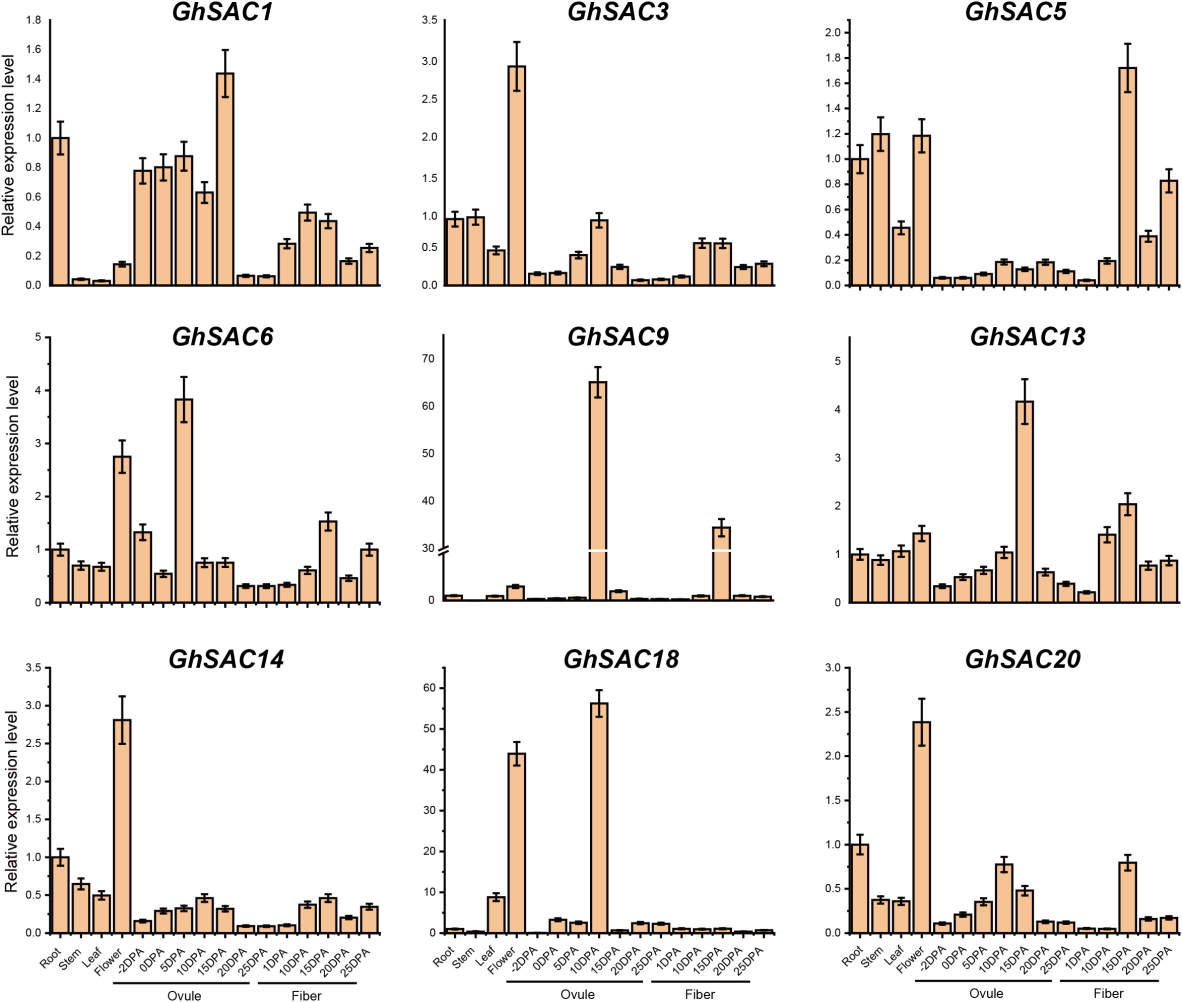
Expression pattern analysis of *GhSAC* genes. qRT-PCR analysis was performed to observe the relative expression patterns of *GhSAC* genes in vegetative, ovule, and fiber tissue of the cotton plant. Each experiment was conducted in three biological repeats and the error bar represents the standard deviation among repeats.

The biological functions of genes are generally correlated with the gene expression pattern. We investigated the transcript level of *GhSAC* genes in various tissues including root, stem, leaf, flower, -2, 0, 5, 10, 15, 20, and 25 DPA ovule, and 1, 10, 15, 20, and 25 DPA fiber (**Figure 5**). Results of qRT PCR analysis displayed that nine selected genes showed ubiquitous expression in all observed tissues. More precisely, *GhSAC1* showed high enrichment in 15DPA ovule tissues. *GhSAC3*, *GhSAC14*, and *GhSAC20* were preferentially expressed in flower tissues, and *GhSAC5* and *GhSAC6* were preferentially expressed in 15DPA fiber and 5DPA ovule. Interestingly, *GhSAC9* and *GhSAC18* were expressed specifically in the 10DPA ovule. Overall, three *GhSAC* genes (*GhSAC3*, *GhSAC14*, and *GhSAC20*) showed preferential expression in flower tissues, while five *GhSAC* genes (*GhSAC1*, *GhSAC6*, *GhSAC9*, *GhSAC13*, and *GhSAC18*) showed preferential expression in ovule tissues. However, only one *GhSAC* gene (*GhSAC5*) showed preferentially expressed in fiber tissues. From these findings, we may infer that *GhSAC* genes might play a significant role in flowering and fiber development in cotton.

### Responses of *GhSAC* genes under abiotic stresses

**Figure 6:**
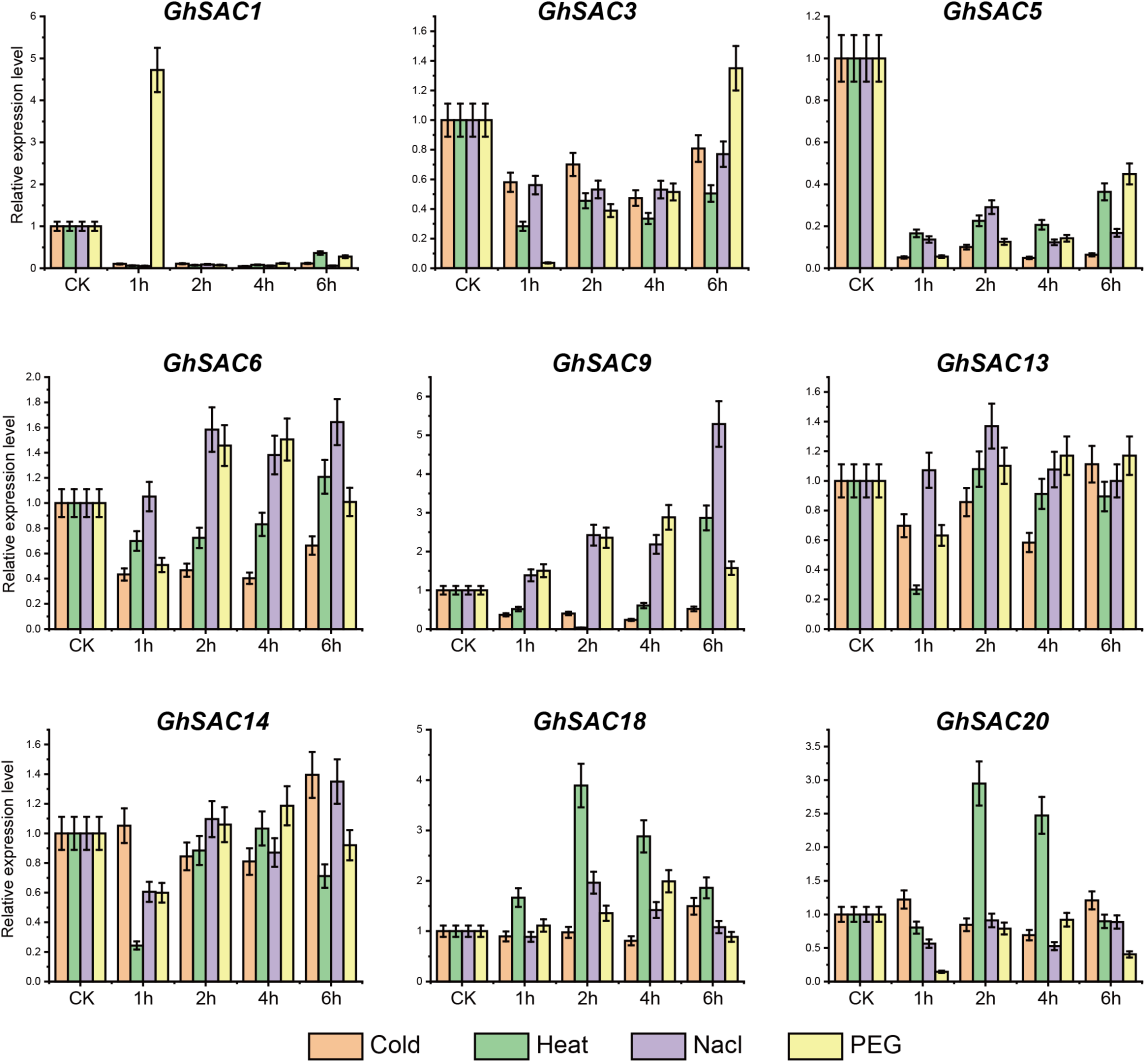
Responses of *GhSAC* genes under abiotic stresses. qRT-PCR analysis was performed to observe the relative expression patterns of *GhSAC* genes under cold, heat, NaCl, and PEG treatment. Each experiment was conducted in three biological repeats and the error bar represents the standard deviation among repeats.

To check the potential biological and physiological function of *GhSAC* genes, we performed the tissue specific expression pattern of nine *GhSAC* genes under various stresses including cold, heat, NaCl, and PEG (**Figure 6**). Abiotic stresses regulate the expression pattern of various genes and affect plant growth and development. *GhSAC* genes showed widely variable responses against all stresses. Overall, *GhSAC* genes showed downregulation under various abiotic stresses except at a few time points for some abiotic stresses. For instance, *GhSAC5* was downregulated under all stresses at all time points, while *GhSAC1* and *GhSAC3* were upregulated only at 1h and 6h after PEG treatment respectively. More precisely, *GhSAC6* and *GhSAC9* were upregulated under NaCl treatment at all time points, while *GhSAC9* and *GhSAC18* were upregulated at all time points under PEG and heat stress respectively. However, most of the *GhSAC* genes did not show any specific pattern of upregulation or downregulation at different time points under any specific abiotic stress treatment. Taken together these findings suggest that the transcript level of *GhSAC* genes can be regulated by various abiotic stresses illustrating that these might be the possible candidate genes for breeding stress resistance in cotton.

## Discussion

Allotetraploid cotton including *G. hirsutum* and *G. barbadense* are the result of hybridization between A (*G. herbaceum* or *G. arboreum*) and D (*G. raimondii*) genome diploid cotton (Wendel and Cronn, 2003). Availability of cotton genome sequences enabled the researchers to perform the evolutionary and functional analysis of various gene families. Functions of *SAC* genes have been previously identified in *Arabidopsis*. *SAC* genes are essential for the phosphoinositide phosphatase activities in animals and yeast (Zhong and Ye, 2003). But there is no previous study of *SAC* genes in cotton, especially *G. hirsutum*. Previously, many gene families including *MADS-box* (Ren et al., 2017), *GhKLCR1* (Li et al., 2019), *RH2FE3* (Qanmber et al., 2018), *GhGSK* (Wang et al., 2018), *GhGH3* (Yu et al., 2018), *GhBES1* (Liu et al., 2018), *GhIDD* (Faiza et al., 2019), *GhAAI* (Qanmber et al., 2019c), *GhHH3* (Qanmber et al., 2019a), *GhPERK* (Qanmber et al., 2019b), *GGPPS* (Ali et al., 2020), *GhGATL* (Zheng et al., 2020), *GhLOG* (Wang et al., 2021), and *GhPHD* (Wu et al., 2021b) has been studied. In the present study, we conducted a complete investigation of the *SAC* genes in eight cotton species. Evolutionary relationship through phylogenetic analysis, sequence logos analysis, gene structure, protein motifs, chromosomal localization, gene duplication, multiple synteny, and collinearity analysis was determined. *GhSAC* gene functions were also observed by *cis*-element analysis, tissue specific expression pattern analysis, and response of *GhSAC* genes under abiotic stresses.

### Evolution of *SAC* genes in cotton

*SAC* genes in eight cotton species including *G. arboreum*, *G. hirsutum*, *G. herbaceum*, *G. raimondii*, *G. darwinii*, *G. barbadense*, *G. mustelinum*, and *G. tomentosum*, could be categorized into five groups through phylogenetic analysis. We found that SAC-d was the largest group containing 44 members while SAC–e was the smallest group with 12 members. The phylogenetic tree indicated that all groups namely SAC- a, SAC- b, SAC- c, and SAC- d contained *SAC* genes from eight observed species while SAC-e lacked the genes from *G. raimondii.* The presence of *SAC* genes in each observed species, with the highest number of *SACs* in *G. mustelinum* and only 10 *SAC* genes in *G. herbaceum* indicates that *SAC* genes have more expansion in plants. These results were coherent with the sequence logos of *G. arboreum*, *G. hirsutum*, *G. herbaceum*, *G. raimondii*, *G. darwinii*, *G. barbadense*, *G. mustelinum*, and *G. tomentosum* that were conserved in all selected *Gossypium* species, demonstrating that *SAC* gene family is conserved throughout the evolution. The number of *SAC* genes in tetraploid species *G. hirsutum*, *G. mustelinum*, *G. barbadense*, *G. tomentosum*, and *G. darwinii* was equal to the total of *SAC* genes in diploid cotton species *G. herbaceum, G. arboreum* and *G. raimondii*, which prove that the tetraploid (AD genome) cotton species formed from diploid A- and D-genome ancestors (Wendel, 1989; Wendel and Cronn, 2003).

Structural analysis of *GhSAC* genes indicated that they have multiple numbers of exons and introns. Structural differences of exon–intron is the result of insertion or deletion and are very important for understanding the evolution of gene families (Lecharny et al., 2003). During evolutions, introns showed weak selection. Loss or gain of introns during eukaryotic diversification was extensive as proved by different genome-wide studies (Rogozin et al., 2003; Roy and Penny, 2007). During the evolution of plant species, introns played a significant role (Roy and Gilbert, 2006). Different length of introns among genes demonstrated their major roles in the functional divergence of *GhSAC* genes. Further, 10 conserved protein motifs were found in GhSACs with slight protein motif differences that might be related to plant growth and abiotic stress tolerance. Results of protein motif analysis showed the specificity of some motifs to a particular group, signifying the characteristic functions of that group.

Chromosomal location showed that *GhSAC* genes were distributed unevenly on different chromosomes. Uneven allocation of *GhSAC* genes on the A and D subgenome chromosomes indicated gene addition or deletion as a result of WGD or segmental duplication events as well as due to incomplete genome sequencing. Most of chromosomes such as A04, A05, A07, A09, A13, D02, D03, D04, D05, D07, D09 and D13 have only one gene. A maximum number of genes (three *GhSAC* genes) were found on A02 and two *GhSAC* genes on D06 and D10 chromosomes. Furthermore, *GhSAC* genes contained various *cis*-elements in their promotor region related to light responsive, zein metabolism, circadian control, phytochrome regulation elements, anaerobic induction, low temperature, meristem expression, and endosperm expression, auxin response, salicylic acid response, abscisic acid response, MeJA response, and gibberellin response elements. Previous studies found light-induced *cis*-elements G-box, GT1-motif, I-box, and AT-rich regions (Lam and Chua, 1989; Gilmartin et al., 1992; Foster et al., 1994), auxin-induced *cis*-elements AuxRE, DR5 (Ulmasov et al., 1997), drought-induced *cis*-elements CATGTG and CACG (Tran et al., 2004). The existence of different elements in the promotor region of *GhSAC* genes predicted the functional diversity of *GhSAC* genes in cotton.

### Duplication and expansion of *SAC* genes

*G. hirsutum* is used to study polyploidy in plants. Previous studies proved that *G. hirsutum* was formed by the hybridization of *G. arboreum* and *G. raimondii* (Wendel, 1989). During the process of evolution, segmental duplication and translocation are known as chromosome mutation help plants to adapt to environmental stresses (Fraser et al., 2005). In our study, the evolutionary mechanism of *SAC* genes was not fully revealed by phylogenetic analysis, therefore we explored genomic distribution and duplication. We observed that the total number of *GhSAC* genes was equal to the total of *SAC* genes in *G. arboreum* and *G. raimondii*. Here, segmental or WGD was the key to *SAC* gene family extension during evolution. Some previous studies also demonstrated gene family expansion through segmental duplication (Qanmber et al., 2018; Wu et al., 2019; Zhao et al., 2020; Wu et al., 2021b).

In this study, *G. arboreum*, *G. herbaceum*, *G. raimondii*, *G. hirsutum*, *G. barbadense*, *G. tomentosum*, *G. mustelinum* and *G. darwinii SAC* genes showed WGD or segmental duplication, however, eight *SAC* genes with the dispersed type of gene duplication and two *SAC* genes with singleton type of gene duplication were also observed. Interestingly, *GhSAC* genes showed only WGD or segmental duplication. Multiple synteny analysis showed almost similar orthologous genes between tetraploid *G. hirsutum* and diploid *G. arboreum*, *G. herbaceum*, and *G. raimondii* (41, 43 and 44 gene pairs respectively) and between *G. hirsutum* and *G. barbadense*, *G. tomentosum*, *G. mustelinum* and *G. darwinii* (65, 65, 64 and 66 gene pairs respectively). *Ka/Ks* ratios among all orthologous gene pairs were less than one indicating the purifying selection of duplicated genes. Next, the locus relationship among A- subgenomes and D-subgenomes chromosomes of *G. hirsutum* and *G. barbadense* showed 10 orthologous/paralogous *GhSAC* gene pairs in *G. hirsutum* with *Ka/Ks* < 1, and 28 orthologous/paralogous genes in *G. barbadense* with *Ka/Ks* < 1. The *Ka/Ks* ratio provides insights into the pressure of selection experienced by duplicated genes during the course of evolution. *Ka/Ks* = 1.0 represents a neutral selection of duplicated pairs of genes, *Ka/Ks* < 1.0 exhibits purifying selection, and *Ka/Ks* > 1.0 shows positive selection during accelerated evolution. Coupled with these findings we summarized that cotton *SAC* genes experienced WGD or segmental duplication with purifying selection pressure during evolution.

### Expression profile analysis of *GhSAC* genes

Several studies demonstrated that SAC proteins have conserved amino acid motifs essential for the phosphoinositide phosphatase activities in animals and yeast. Gene expression analysis of *Arabidopsis* SAC proteins verified the differential expression of *AtSAC* genes in various organs. More specifically, the *AtSAC6* gene was primarily expressed in flowers and was highly induced by salinity stress (Zhong and Ye, 2003). Here, the expression level of *GhSAC* genes was examined in different vegetative and reproductive organs. Expression profile analysis of *GhSAC* genes displayed that they play important functions in plants. Likewise, *Arabidopsis SAC* genes, and *G. hirsutum SAC* genes exhibited differential expression in various organs and tissues. Three *GhSAC* genes (*GhSAC3*, *GhSAC14*, and *GhSAC20*) showed preferential expression in flower tissues, five *GhSAC* genes (*GhSAC1*, *GhSAC6*, *GhSAC9*, *GhSAC13*, and *GhSAC18*) showed preferential expression in ovule tissues and only one *GhSAC* gene (*GhSAC5*) showed preferential expression in fiber tissues. Here, *GhSAC6* showed increased transcript levels in all observed tissues and organs specifically high expression in flower and 5DPA ovule. These findings are similar to the previous study as the *AtSAC6* gene in *Arabidopsis* was preferentially expressed in flowers and other organs (Zhong and Ye, 2003). Previous studies of *SAC* genes in *Arabidopsis* indicated that *AtSAC* genes showed lower expression in leaves and roots (Zhong and Ye, 2003), but here we observed that all *GhSAC* genes showed moderate to low expression levels in leaves and roots. From these findings, we may infer that *GhSAC* genes might play a vital role in flowering and ovule development in cotton plants.

The *cis*-elements analysis showed that *GhSAC* genes can be regulated by abiotic stress and participate in hormone signal transduction, so we validated these results with the help of expression pattern analysis of *GhSAC* genes in response to different stress stimuli. *SAC* genes have a key role in the phosphatase activities of animals and yeast (Zhong and Ye, 2003). Phosphoinositides metabolism of plants is mainly regulated by different stress treatments and hormones (Mikami et al., 1998; Meijer et al., 1999; Pical et al., 1999; Meijer et al., 2001). So, we explored the expression patterns of *GhSAC* genes under abiotic stress treatments. The transcript level of the *AtSAC6* was induced by salt stress treatment (Zhong and Ye, 2003) demonstrating that *AtSAC6* can be regulated by salt stress. Consistent with the previous studies *GhSAC6* showed high expression under salt treatment. Further, hyperosmotic or salt treatment changes the phosphoinositide level in plants (Meijer et al., 1999; Pical et al., 1999; Dewald et al., 2001). Overall, all *GhSAC* genes showed response under observed abiotic stresses for various time points. For instance, *GhSAC5* showed downregulated response under all stresses, while *GhSAC1* and *GhSAC3* showed upregulated response only at 1h and 6h after PEG treatment respectively. Moreover, *GhSAC6* and *GhSAC9* showed upregulated response under NaCl treatment, while *GhSAC9* and *GhSAC18* showed upregulated response under PEG and heat stress treatment respectively. These findings illustrated that the transcript level of *GhSACs* can be regulated by different abiotic stresses indicating that *GhSAC* genes can be the possible candidate genes for breeding abiotic stress resistance in cotton.

## Conclusion

Here, a total of 157 *SAC* genes were found in eight species of cotton including 23 genes in *G. hirsutum*. Based on the phylogenetic tree *SAC* genes were classified into five distinct groups. WGD or segmental duplication was an important source for the enlargement of the *SAC* gene family in cotton. Cotton *SAC* duplicated genes experienced purifying selection pressure and showed conserved amino acid sequence logos during evolution. *GhSAC* genes showed conserved gene structure with multiple exons/introns and protein motifs. *GhSAC* genes showed uneven chromosomal distribution patterns on different chromosomes of A- and D-subgenomes. *GhSAC* genes play essential regulatory roles in the growth of the cotton plant and can be regulated under abiotic stresses. Based on expression patterns, *GhSAC* genes were associated with flower, ovule, and cotton fiber development. Further, *GhSAC* genes were regulated by abiotic stresses. For instance, three *GhSAC* genes showed enrichment in flower tissues, five *GhSAC* genes were highly expressed in ovule tissues and one *GhSAC* gene was highly expressed in fiber tissues. Similarly, *GhSAC5* was downregulated under all abiotic stresses, *GhSAC1* and *GhSAC3* were upregulated at 1h and 6h after PEG treatment respectively, *GhSAC6* and *GhSAC9* were upregulated under NaCl treatment, and *GhSAC9* and *GhSAC18*weres upregulated under PEG and heat stress respectively. Our study provides useful information related to the evolution of the cotton *SAC* gene family, biological functions of *GhSAC* genes and laid the foundation for further studies of *SAC* genes in other plant species.

## Data availability statement

The datasets presented in this study can be found in online repositories. The names of the repository/repositories and accession number(s) can be found in the article/**Supplementary Material**.

## Author contributions

MS, LL, SH, GY, and LY conducted the experiments. GQ planned the study and conducted the image analysis. All authors contributed to the article and approved the submitted version.

## Supplementary material

Supplementary Figure 1 Sequence logos analysis of cotton *SAC* genes. Amino acid sequence residue analysis was performed among **(A)** *G. herbaceum*, **(B)** *G. arboreum*, **(C)** *G. raimondii*, **(D)** *G. hirsutum*, **(E)** *G. barbadense*, **(F)** *G. tomentosum*, **(G)** *G. mustelinum* and **(H)** *G. darwinii SAC* genes across N and C terminals.

Supplementary Figure 2 Gene structure and protein motif analysis of *GhSAC* genes. **(A)** Phylogenetic analysis among *GhSAC* genes. **(B)** Protein motifs distribution pattern analysis among *GhSAC* genes. **(C)** CDs, introns, and UTR structure of *GhSAC* genes.

Supplementary Figure 3 Chromosomal localization of *GhSAC* genes. *GhSAC* genes were localized on their corresponding chromosomes and the color of chromosomes represents the gene density on that chromosome. A02 to A13 represent the A-subgenome chromosomes of *G. hirsutum* and D02 to D13 represent D-subgenome chromosomes of *G. hirsutum*.
