# Supplementary figures and images for: Genomic identification of cotton SAC genes branded ovule and stress-related key genes in Gossypium hirsutum
Source: Front Plant Sci. 2023 Feb 3;14:1123745. doi: 10.3389/fpls.2023.1123745 (PMC9935941; doi:10.3389/fpls.2023.1123745)

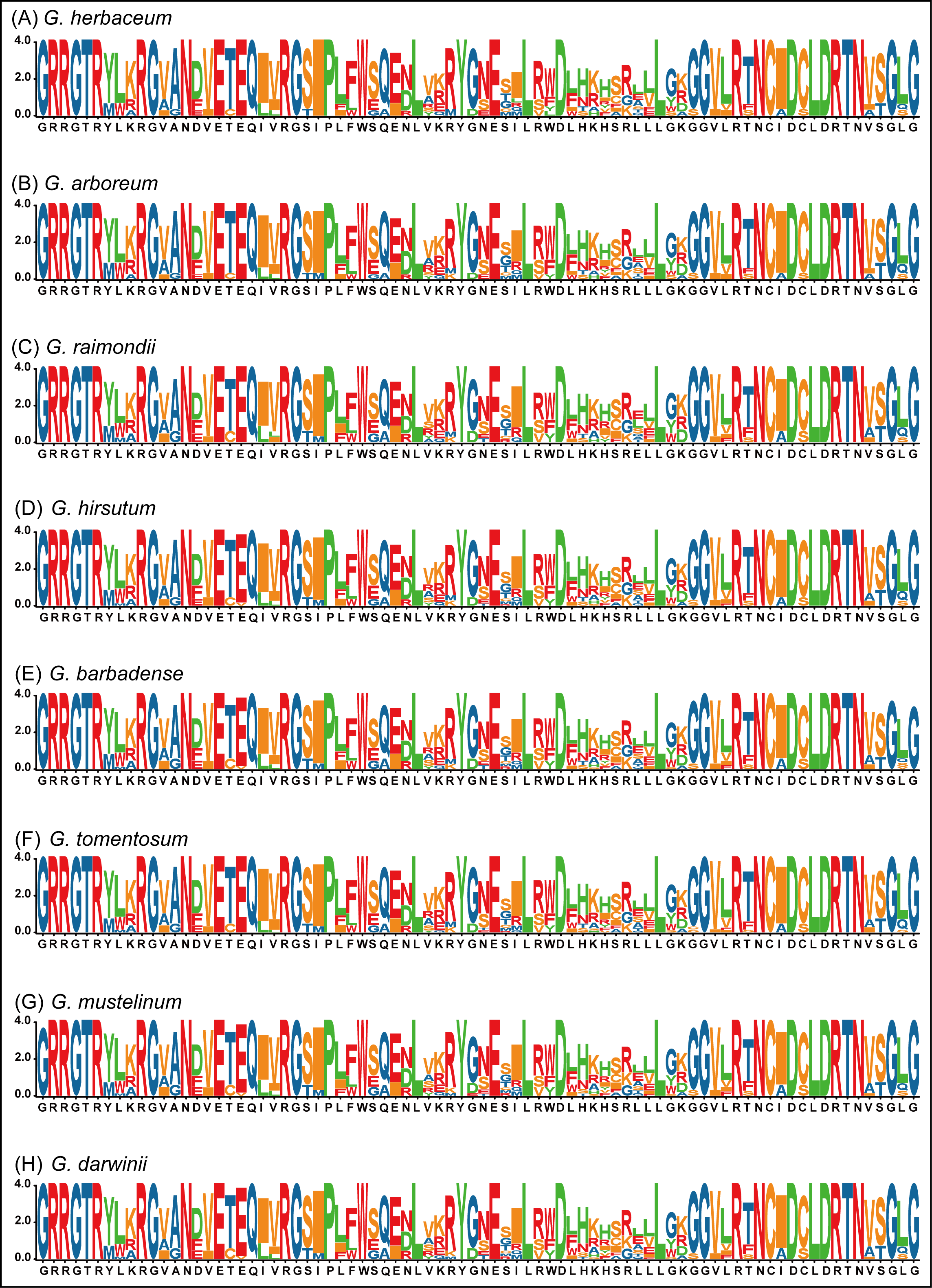

Supplement: Supplementary Figure 1 — Sequence logos analysis of cotton SAC genes. Amino acid sequence residue analysis was performed among (A) G. herbaceum, (B) G. arboreum, (C) G. raimondii, (D) G. hirsutum, (E) G. barbadense, (F) G. tomentosum, (G) G. mustelinum and (H) G. darwinii SAC genes across N and C terminals. [file Image_1.tif]

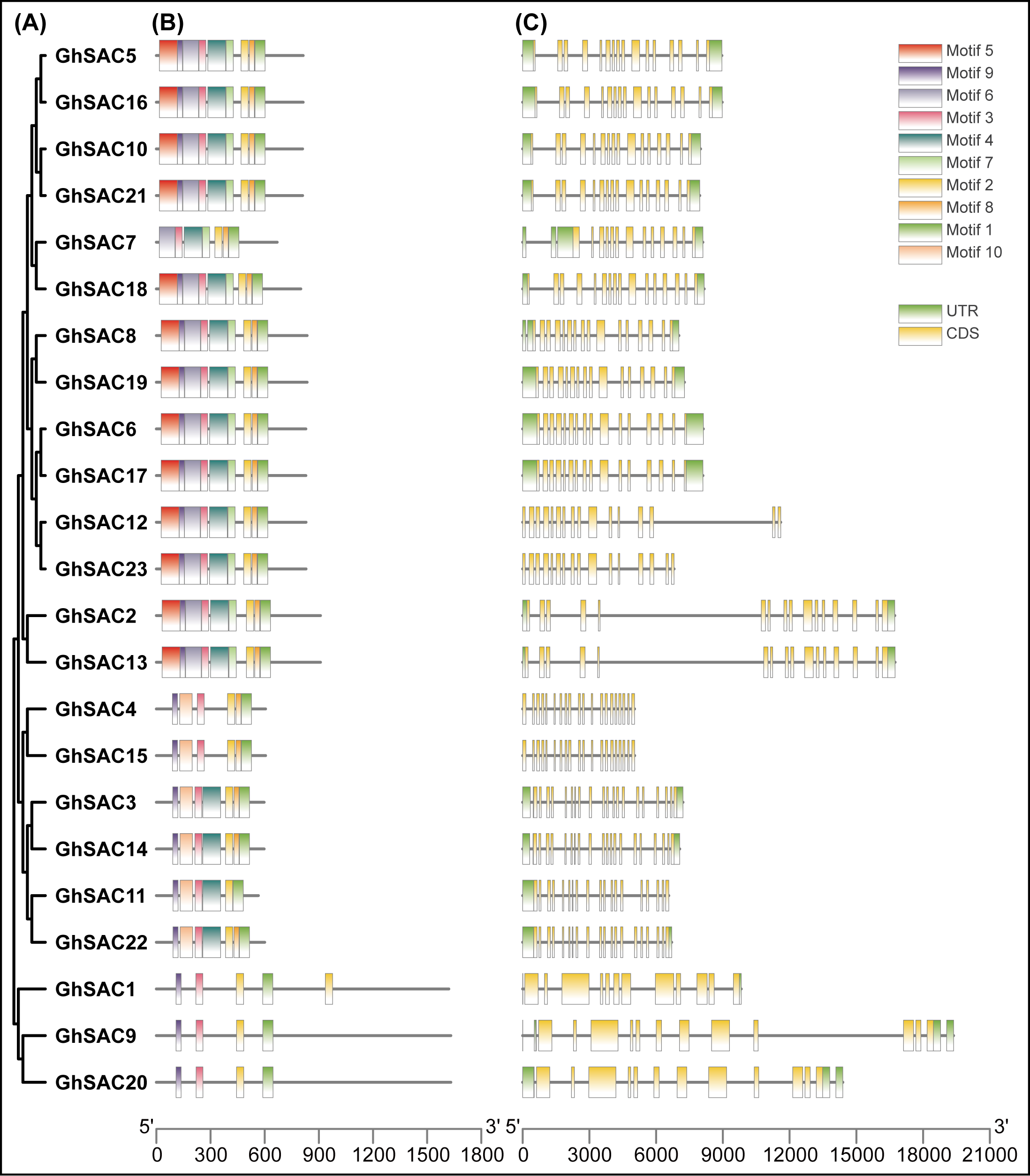

Supplement: Supplementary Figure 2 — Gene structure and protein motif analysis of GhSAC genes. (A) Phylogenetic analysis among GhSAC genes. (B) Protein motifs distribution pattern analysis among GhSAC genes. (C) CDs, introns, and UTR structure of GhSAC genes. [file Image_2.tif]

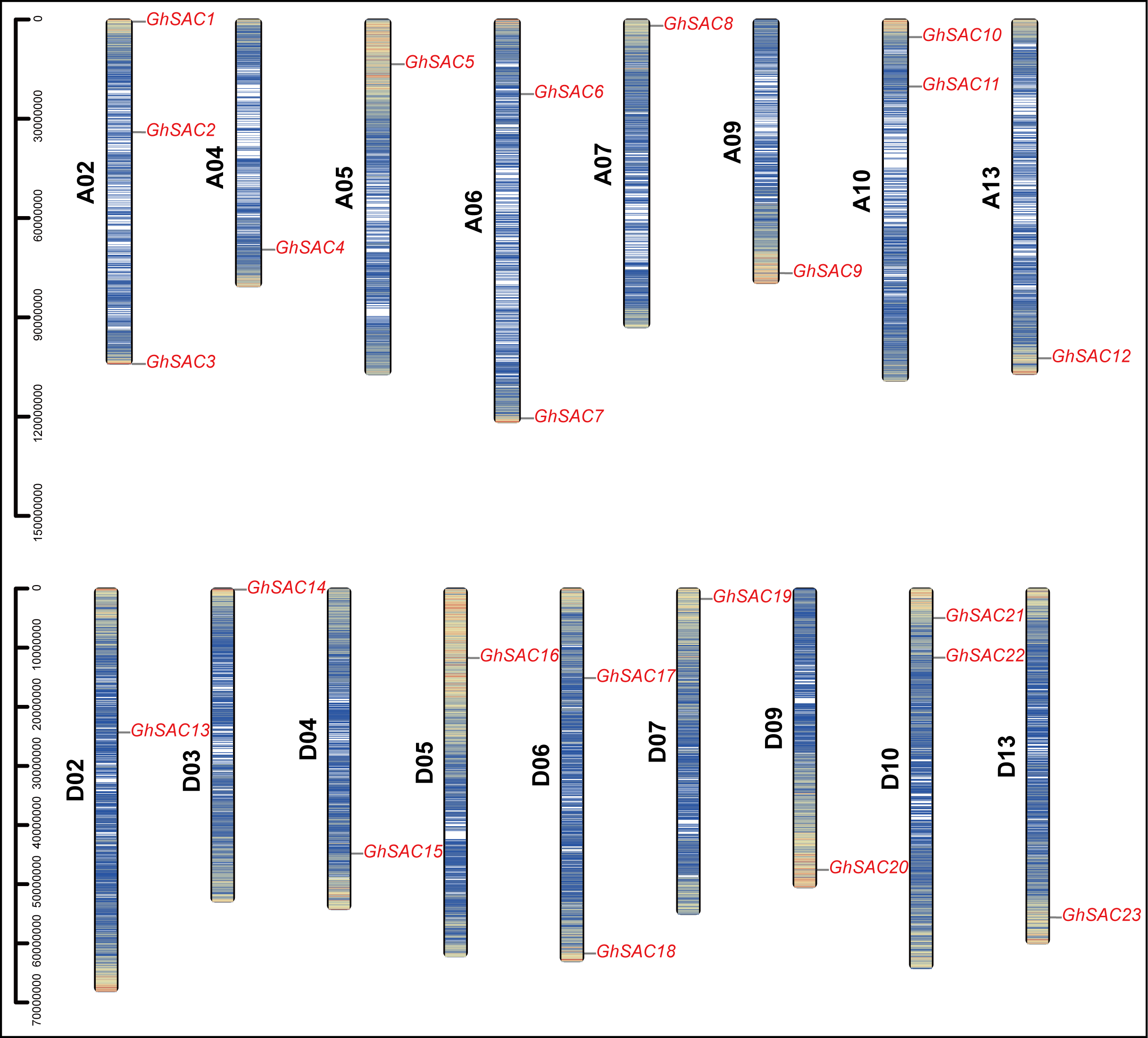

Supplement: Supplementary Figure 3 — Chromosomal localization of GhSAC genes. GhSAC genes were localized on their corresponding chromosomes and the color of chromosomes represents the gene density on that chromosome. A02 to A13 represent the A-subgenome chromosomes of G. hirsutum and D02 to D13 represent D-subgenome chromosomes of G. hirsutum. [file Image_3.tif]
